# The Challenge of Managing Undernutrition in Older People with Frailty

**DOI:** 10.3390/nu11040808

**Published:** 2019-04-10

**Authors:** Helen C. Roberts, Stephen E. R. Lim, Natalie J. Cox, Kinda Ibrahim

**Affiliations:** 1 Academic Geriatric Medicine, Faculty of Medicine, University of Southampton, Southampton SO16 6YD, UK; S.E.Lim@soton.ac.uk (S.E.R.L.); N.Cox@soton.ac.uk (N.J.C.); K.Ibrahim@soton.ac.uk (K.I.); 2 NIHR Southampton Biomedical Research Centre, University of Southampton and University Hospital Southampton NHS Foundation Trust, Southampton SO16 6YD, UK; 3 NIHR Collaboration for Leadership in Applied Health Research and Care (NIHR CLAHRC) Wessex, University of Southampton, Southampton SO16 7NP, UK

**Keywords:** malnutrition, older, frail, screening, appetite, mealtime, food fortification, nutritional supplement

## Abstract

Many older people with frailty are at risk of malnutrition and poor health, yet there is evidence that improving nutrition and weight loss can reduce frailty. This will become more important as the number of older people with frailty increases worldwide in future. Identifying those at risk is challenging due to the difficulty of reaching and screening those older people most at risk, the large number of nutritional assessment tools used, and the lack of consensus on the criteria to make a diagnosis of malnutrition. The management of older people with or at risk of malnutrition should be multi-modal and multi-disciplinary, and all care staff have an important role in delivering appropriate nutritional advice and support. This paper will highlight a number of practical approaches that clinicians can take to manage malnutrition in older people with frailty in community and acute settings, including environmental changes to enhance mealtime experience, food fortification and supplementation.

## 1. Introduction

Under nutrition (herein referred to as malnutrition) is common among older people and often poorly recognised and under diagnosed. It is associated with frailty, sarcopenia and poor health outcomes. Importantly, there is increasing interest in the potential of nutritional interventions to improve the poor nutrition and weight loss of frailty, but current evidence is scarce [1]. 

The current definition of malnutrition from the European Society for Clinical Nutrition and Metabolism (ESPEN) is ‘a state resulting from a lack of uptake or intake of nutrition leading to altered body composition (decreased fat free mass and body cell mass) leading to diminished physical and mental function and impaired outcome from disease’ [2]. Malnutrition is often defined as inadequate intake of dietary energy and protein. ESPEN recommends a guiding value of 30 kcal/kg body weight/ day for people aged >65 years, to be adjusted for gender, nutritional status, disease state and physical activity [3]. Protein intake of at least 1.0 g/kg/day is recommended by ESPEN for older people to maintain muscle mass, increasing to 1.2–1.5 g/kg/day for those with acute or chronic illness to support wound healing, immune function and recovery [4]. Micronutrients are also important and frail older people with apparently normal nutritional status are commonly deficient in micronutrients such as vitamin D (termed qualitative malnutrition) [5].

It has been estimated that 3 million people in the United Kingdom (UK) alone are poorly nourished or at risk of becoming so, with most (93%) living at home [6]. The prevalence of malnutrition among older people varies by setting and has been reported in a meta-analysis of 240 studies as 3% among community dwelling older people, 6% among those attending outpatients and 8.7% among those receiving home care services, but with considerable heterogeneity between studies [7]. Malnutrition is highly prevalent in hospitals and a pooled analysis of data from older people aged 65 years and over in hospitals in 12 countries reported that 39% were at risk of malnutrition [8]. Nutritional status is estimated to worsen during hospital admission among 60% of older people [9] for a variety of reasons including the effects of acute illness [10], cognitive impairment due to delirium and dementia, low mood, medications, comorbidities, poor dentition, dysphagia [11] and poor appetite [12]. The prevalence of malnutrition in care homes is also high with estimates ranging from 14% [8] to 30% [13]. 

Frailty is often defined as a loss of biological reserves across multiple organ systems with increased vulnerability to physiological decompensation after a stressor event [1]. Frailty is associated with poor health outcomes including increased disability, admissions to hospital and care homes, and mortality. The prevalence of frailty varies by setting and is reported to be 9–10% among community dwelling older people [14,15] with around 45% classified as ‘pre-frail’. Frailty is more common with increasing age, affecting around 25% of people aged >85 years [16], with high prevalence among older hospital patients and in nursing homes (52% in a pooled estimate of 1373 people aged ≥60 years) [17]. 

Malnutrition and frailty are related syndromes [18]. Many frailty measures include weight loss, and indeed weight loss is postulated to be a modifiable factor in frailty [19]. A recent meta-analysis of 5447 community dwelling older adults including out-patients (mean age 77 years) from 10 studies in Belgium, Germany, Spain, Turkey, Mexico, Russia and Japan identified 2.3% as malnourished using the Mini-Nutritional Assessment and 19.1% as physically frail using the Fried Frailty Phenotype [20]. Among those who were malnourished, 68% were also frail while only 8.4% of those with physical frailty were also malnourished. Similar findings have been reported among older community dwelling people in Singapore with an additional association between pre-frailty and malnutrition or risk of malnutrition [21]. Malnutrition is also strongly associated with sarcopenia (loss of muscle strength, function and mass with increasing age) which leads to reduced physical function and dependency. Low muscle mass is central to the diagnosis of malnutrition and sarcopenia [22] although it can be challenging to measure in routine clinical practice [23].

The causes of malnutrition in older people are multifactorial [3]. Reduction in smell and taste sensations, poor appetite (anorexia of ageing), and altered dietary choice and habits contribute to insufficient dietary intake. Acute illness and medications can exacerbate anorexia, as can poor dentition (such as tooth loss, and poorly fitting dentures), visual impairment and gastrointestinal conditions. Nutrition among older people may be impacted by living or eating alone, caring for their partner, access to shops, ability and motivation to cook, illness and low income [24,25]. Psychological factors including loneliness, dementia and depression are recognised to have a negative impact on the dietary intake of older people in all settings [26]. 

Malnutrition has serious consequences for individuals, society, health, and social care services. Malnutrition is associated with increased risk of frailty, sarcopenia, falls, dependence in activities of daily living (ADL), hospital admission and longer length of stay, with poor wound healing and more complications, increased mortality and poor health-related quality of life [3]. Malnutrition is associated with increased use of primary care services [27] and a study of community dwelling older Canadians reported 20% higher odds of hospital admission and 60% higher odds of death over 3 years among those at risk of poor nutrition [28]. Among a cohort of 800 Colombian hospital patients, malnutrition was associated with 30% higher costs of hospital stay [29]. Malnutrition was estimated to cost £19.6 billion in the UK alone in 2011–2012 in health and social care costs [30]. Malnourished patients diagnosed whilst living at home in the UK are reported to have healthcare costs that are twice as much as those of non-malnourished patients [31]. 

There are a number of practical approaches to the management of malnutrition in frail older people that can be undertaken, starting with early identification of those at risk. 

## 2. Screening and Identification of Malnutrition

### 2.1. The Challenge of Screening and Diagnosis

The diagnosis of malnutrition can be made following a formal nutritional assessment. However, there has long been a lack of consensus regarding the criteria needed to make a diagnosis of malnutrition. Ongoing work by the Global Leadership Initiative on Malnutrition has recently produced a statement on diagnostic parameters which focuses on intentional weight loss, low body mass index (BMI) and reduced muscle mass as the three phenotypic criteria, and reduced food intake or absorption and inflammation or disease burden as the two aetiologic criteria (Table 1) [32]. 

For a diagnosis of malnutrition, at least one of each set of criteria needs to be met. A grading of severity can then be made based on thresholds for the phenotypic criteria (Table 2). 

Nutritional assessment tools such as the Subjective-Global Assessment (SGA) and the Mini-Nutritional Assessment (MNA) aid in diagnosis. However, use of these tools in older people is supported by little evidence [33]; diagnosis in this group is further complicated as BMI is potentially an inaccurate measure for predicating outcomes [34] and weight and height of patients are still not systematically recorded in many hospitals [35]. Measuring muscle mass can also be challenging and one study reported that it was only feasible among 50% of older inpatients [23]. 

Before formal assessment occurs, a person is often identified to be at ‘nutritional risk’ by a malnutrition screening tool [32]. Current recommendations for screening older people are determined by setting with yearly screening in the community, directly on any hospital admission and screening every three months in care homes [3]. There are a number of screening tools available and ESPEN recommends the use of the Nutritional Risk Screening-2002 (NRS-2002), MNA- short form (MNA-SF) and the Malnutrition Universal Screening Tool (MUST); with a focus on the MNA-SF for older people [36]. However, a recent review of malnutrition screening tools used for older people across settings reported 48 different tools in use, of which 36 were validated in people aged ≥65 years [37]. The large number of tools available obscures the true prevalence of malnutrition across older populations and contributes to the variability in reported rates [2].

The use of validated malnutrition screening tools in hospital is associated with increased monitoring of oral intake, with algorithms for addition of supplements and dietician referral that can provide individualized nutritional support [38], to improve clinical and functional outcomes [39,40,41]. Reported barriers to implementing screening include a lack of organisational culture recognising its importance, lack of staff education and training, and staff perceptions that clinical judgement is more pertinent than the use of tools [42]. Despite these barriers, awareness and implementation of routine malnutrition screening on admission to hospital is improving [43].

The rise in hospital screening is currently not reflected in the community, despite policy recommendations, and there is a lack of research on attributable clinical outcomes in this setting [27,44,45]. This is problematic considering the high numbers of older people with frailty in the community, and has led to a call for high quality research into the effectiveness of nutritional screening in this setting [46]. There are currently initiatives within the UK that may go some way to answering the question of how to implement large-scale screening and treatment programmes for malnutrition in community dwelling older people [47,48,49].

### 2.2. Screening for Malnutrition in Different Settings

#### 2.2.1. Community

The most commonly used screening tool in studies of malnutrition among community dwelling older people is the MNA-SF [37]. However, the MNA-SF is consistently validated against MNA itself, holding inherent bias in the interpretation of its favourable sensitivity and specificity in identifying malnutrition [37]. Other widely assessed screening tools in community dwelling older people are Determine Your Health Checklist (DETERMINE) and Seniors in the Community: Risk Evaluation For Eating And Nutrition Questionnaire (SCREEN-II) tools [37]. DETERMINE performs poorly at identifying malnutrition and is likely to overestimate nutritional risk in this setting [37]. SCREEN-II performs better in the community with good validity against dietician assessment and so could be considered preferable in this setting [37].

#### 2.2.2. Hospital Setting

Recently, Cascio et al. [50] appraised the most frequently used malnutrition screening tools for hospitalised older people: the MNA-SF, MUST, Malnutrition Screening Tool (MST), the NRS-2002 and the Geriatric Nutrition Risk Index [50]. On comparison, no tool proved most effective within the limitations of heterogeneity of included studies; all demonstrated similar abilities at identification except the MNA-SF which tended to overestimate malnutrition risk [50]. In the hospital setting [37], the validity of MUST and MST were supported by a number of studies in older people across different countries with the authors also reporting that the MNA-SF can overestimate malnutrition risk. Thus, the MUST and MST, although not created for older people, could be considered the most appropriate tools for older people in hospital [37].

#### 2.2.3. Care Homes

A systematic review of malnutrition screening within the care home setting assessed over 20 different tools used and reported the validity of the best performing tools to be only fair [51]. The MNA-SF was often used but when validated against alternatives to the MNA it performed poorly [51]. The MUST has also been used frequently but performs fair to poor in this setting [37,51]. The Short Nutritional Assessment Questionnaire for Residential Care (SNAQ-RC) demonstrated fair validity in the residential setting so could be considered a preferable tool [37], however there is in general little evidence for the use of any of the current malnutrition screening tools in the care home population [51].

### 2.3. Screening for Appetite Loss

Loss of appetite, or the Anorexia of Ageing, is a known major contributor to malnutrition and may be measurable prior to weight change [3,32,52]. A recent systematic review [53] of current methods to assess appetite in older people when determining the efficacy of potential interventions reported a lack of consensus of how to assess appetite. Achieving a standardised approach to the assessment of appetite may be a useful route to identify those at risk of subsequent weight loss.

## 3. Nutritional Education and Advice

The management of older people with or at risk of malnutrition should be multi-modal and multi-disciplinary [3]. Care staff have an important role in delivering appropriate nutritional advice and support, and making timely referrals for specialist dietetic advice in line with local care pathways. For example, older people with tooth loss treated with dentures or dental implants demonstrated a greater improvement in fruit and vegetable consumption when combined with dietary counselling, compared to receiving dental treatment alone [54].

In primary care settings, community healthcare professionals, especially nurses, may be able to deliver opportunistic nutritional advice and education, and there is evidence that this can positively influence the functional outcomes and diet of older people living at home [55]. Group educational sessions can be effective [56] but older people with frailty may find it difficult to access these. Telehealth methods such as telephone consultations, tele-monitoring devices and internet-enabled tablets have been reported to be effective among community dwelling older people in a recent systematic review and meta-analysis [57].

## 4. Environmental Changes to Enhance Mealtime Experience

A range of environmental factors can influence the mealtime experience of older people in different settings. Effective interventions to address these factors could potentially reduce the risk of malnutrition among older people.

### 4.1. Community

Several studies have explored the role of family, friends and neighbours in improving nutrition of community-dwelling older people, as they have a unique insight into their environment, values, traditions and beliefs [56]. A review by Marshall et al. reported moderate evidence to support engagement of family carers as part of the nutrition care team for older adults with malnutrition [56]. A randomised controlled trial conducted by Salva et al. showed a reduction in risk of malnutrition (MNA score, +0.46 vs −0.66, *p* = 0.028) among older adults with dementia when their main caregivers received health and nutrition training, compared to the control group [58]. The nutrition training focused on nutritional monitoring, lifestyle habits, balanced diet and the food pyramid, nutritional support preparations, and nutritional supplements. However, the intervention had no effect on functional decline as measured by Activities of Daily Living (ADL) and Instrumental Activities of daily living (IADL) scales.

Volunteers can also help reduce malnutrition risk among community-dwelling older adults. A randomised controlled trial among 80 older adults (mean age 83 years) reported that peer volunteers trained to perform strength exercises and have nutrition-related discussions with patients resulted in improved MNA scores and a 25% reduction in prevalence of impaired nutritional status (MNA score < 24) [59]. The study also demonstrated an improvement in frailty status among participants who received the volunteer-led physical training and nutritional intervention. Frailty was measured using the Frailty Instrument for Primary Care of the Survey of Health, Ageing and Retirement in Europe (SHARE-FI).

There is evidence that people tend to eat more when dining with others rather than alone [60,61], an effect termed ‘social facilitation’ [62]. Older people are more likely to have better food intake with improved nutritional risk when eating with others [63]. Dining with family and friends creates a relaxed environment, increasing meal duration, leading to a higher level of food intake [61]. Other ambient factors recognised to impact on food intake include food accessibility, room lighting and temperature, smell of food and appropriate music [62]. Among older people who have a small appetite, the size, timing and frequency of meals should be considered [64,65]. It may be better to provide small but frequent opportunities for eating rather than large plates of food which may deter an older person from eating at all. Offering nutritious snacks in between meals may be one way of achieving this [3]. Mealtimes must not be rushed—allowing enough time for people to eat, and offering encouragement to eat may be helpful.

Recognised barriers to eating well among older people living at home include the affordability and accessibility of food, limited food services, decreasing mobility, and lack of cooking skills [66,67]. Local day centres and day services, home shopping services, lunch clubs and community cafes are important [68] especially for those who are frail, have limited mobility, or little access to personal transport [69]. The supply of frozen meals is less well received by older people compared to hot meals [68] but meal delivery programs are reported to improve food intake, social interaction and quality of life, and reduce food insecurity and nutritional risk among participants [70].

### 4.2. Hospital Setting

Malnutrition is common among hospitalised older people and in the busy clinical setting of an acute hospital, multiple environmental factors can influence the nutritional intake of older inpatients. These include mealtime interruptions by medication rounds and other clinical activities, inflexible food service practices, unfamiliar food items, time and staffing shortages, and practical issues such as inappropriate placement of food trays and food packaging that is hard to open [71,72,73,74]. Food delivery systems (pre-plated portions, portioned on the ward, use of microwaves to reheat food) can variably affect food texture, temperature, flavour or condensation. However, it appears that the food journey (mainly temperature changes and condensation) affects only a small number of sensorial descriptors related to flavour, appearance and mouthfeel and is estimated not to substantially contribute to malnutrition in hospital [75]. The pre-plated service is known to be less flexible and requires choice and pre-ordering hours or days before the meals: systems which allow food ordering at mealtime are reported to result in higher food intake, less waste, better use of oral nutritional supplements, and higher patient satisfaction in terms of choice, hunger, food quality and organisation [76].

Several initiatives have been developed to address these issues. Coloured trays have been used to help staff identify those patients who may need help during mealtimes [77]. Although widely adopted, there is a lack of evidence on its impact on nutritional intake [78]. Protected mealtimes have also been introduced to prevent non-urgent clinical activity from interrupting mealtimes to allow ward staff time to offer assistance [79]. The evidence for protected mealtimes is mixed [80]. Some studies have shown that protected mealtimes as a single intervention did not change patients’ mealtime energy and protein intakes [81,82,83]. A pre–post study by Young et al. reported that protected mealtimes and additional feeding assistance (implemented alone or in combination) might produce modest improvement in nutritional intake [84]. Communal dining has also been shown to improve food intake among hospitalised older people [71,85]. However, this has to be interpreted with caution as the ability to get to the dining room may also reflect those patients’ better physical function and recovery from illness. Mealtime positioning is also important, as patients sitting up for their meal are likely to eat more of their meal than those lying in bed [86].

Mealtime assistance can also improve nutritional intake among hospitalised older people. A meta-analysis by Tassone et al. reported statistically significant increases in daily energy (mean difference of 486.4 kJ, 95% CI 11.15–961.66 kJ) and protein (mean difference 5.86 g, CI 1.09–10.63 g) intake in older inpatients receiving mealtime assistance compared to control groups [87]. Studies examining the impact of mealtime assistance have included paid staff members (nurses and nursing assistants) [80,88] and volunteers [89,90]. The use of volunteer mealtime assistants has been shown to have a positive impact on the nutritional intake of older inpatients [91,92] and may free up valuable nursing time with potential cost-savings [93].

### 4.3. Care Homes

A review of the qualitative literature around attitudes, perceptions and experiences of mealtimes among care home residents and staff was conducted by Watkins et al. to better understand factors that may contribute to malnutrition in this setting [94]. One of the major themes identified in the review was insufficient organisational support at mealtimes, with a lack of staff to support during mealtimes who feel pressured to complete other routine tasks [95,96]. Another key theme identified was meal quality and enjoyment relating to the physical aspects of mealtime, including dining environment and atmosphere, meal options, quality of food and type of food service [97,98,99].

Observational studies have shown that one-to-one feeding assistance increased oral intake among care home residents [100,101] and volunteers are increasingly being used to help improve mealtime care among care home residents [102]. However, a systematic review in 2011 reported low level evidence regarding the effectiveness of volunteer mealtime assistants in promoting increased nutritional intake among residents in institutional settings [103].

Several interventions have been explored to address the environmental factors that may increase the risk of malnutrition among older adults in care homes. These include the use of music to alter dining ambience [104], use of high contrast red tableware [105], bulk food delivery service and having a more home-like environment [106,107], family-style meals (food placed in large bowls in the centre of the table for self-service), or buffet-style meals [108,109]. A systematic review by Liu et al. concluded that there was low level evidence of the effectiveness of these environmental modifications on the food intake of older adults with cognitive impairment [110]. A meta-analysis conducted by Abbott et al. explored the effectiveness of mealtime interventions on nutritional outcomes of older people living in residential care and showed some evidence of an effect on daily energy intake with dining environment alterations. [111] However, evidence regarding the impact of dining environment alteration on body weight was inconsistent. Further research in this area is required.

## 5. Food Supplementation and Fortification

### 5.1. Food Fortification

This strategy is based on ‘food first’ principles and involves using energy and protein dense meals (fortification) or snacks (supplementation) to increase dietary calorie and protein intake. Examples include the use of fortified bread [112,113], soups [114], sauces [115], protein and dairy-enriched main meals [116,117], and high-calorie between-meal snacks or desserts, such as biscuits [118], yogurt [112,113] and ice cream [119]. Systematic reviews have reported that energy- and protein-based fortification and supplementation could be employed as an effective, well-tolerated and cost-effective intervention to improve the dietary intake of older people [120,121] especially those with frailty and cognitive impairment. The impact of fortified food on functional and clinical outcomes is little understood as the focus is mainly on nutritional intake. No significant differences were found between the intervention and control groups in relation to body weight, hand grip strength or length of stay from admission [113]. In addition, there is some evidence for the role of flavour enhancement in increasing food intake among older people with frailty. Researchers reported that the addition of both seasoning and fortified sauce to an older person’s meal can increase energy, protein and fat intake [115,122].

### 5.2. Finger Foods

There is evidence that offering “finger foods”—foods that can be easily eaten with fingers—can increase the pleasure of eating, increase food consumption and improve autonomy among older people living with frailty and dementia. A study among care home residents showed that finger foods were frequently chosen and consumed [123]. This strategy was judged by health professionals as an easy, cheap and useful solution [124]. Finger foods may be a simple way to provide nourishment, food enjoyment and the dignity that comes with independence.

### 5.3. Oral Nutritional Supplements

Oral Nutritional Supplements (ONS) in the form of energy- and protein-dense sip feeds can be effective to improve nutritional intake among malnourished older people including those with frailty [125]. ONS are cost effective (mean net cost saving of £746 per inpatient) and are associated with reductions in mortality, readmissions and complications in the acute setting [126,127,128,129]. Similarly, the cost-effectiveness of ONS is well documented among community dwelling older people and care home residents [130], with clinically relevant benefits including improved quality of life and reductions in infections, minor post-operative complications, and falls [125]. A Cochrane review suggested that dietary advice with or without oral nutritional supplements may improve weight, body composition and grip strength but with no evidence of benefit on survival [128]. Nutritional supplements have also been shown to be effective in improving physical performance outcomes in frail or sarcopenic older people [131]. A randomized controlled trial conducted by Bo et al. demonstrated that the combined supplementation of whey protein, vitamin D and E improved muscle mass and muscle strength in older adults with sarcopenia [132]. ONS should be given between meals, as they may contribute to reduced consumption of food when given at mealtimes [133]. Best results are seen when people are offered a variety of different flavours, consistencies and if administered in small regular doses similar to a medicine [134]. It should be noted that the main focus is on protein–energy malnutrition [135], but a recent National Diet and Nutrition Survey (NDNS) in the UK reported that dietary intakes of vitamins D and K, magnesium and selenium among older people living in the community were frequently below recommended nutrient intake values [136]. However, current guidelines do not recommend routine prescription of vitamin and mineral supplements to treat malnutrition unless specific deficiency is identified [3].

However, the acceptability and intake of ONS are frequently suboptimal in older people [137], specifically those living with frailty or dementia, and wastage of up to 35% of these products is reported [138]. This may be because older people do not like the flavour, texture and/or smell of ONS [139] which can offer little sensory variety, and so ‘taste fatigue’ can develop [125]. Poor concordance may be exacerbated by delirium and cognitive impairment and may be mediated by a lack of familiarity with sip feeds [140]. Older people are particularly reliant on visual cues when judging flavour and food liking, and may not accept unfamiliar food items [141,142]. A systematic review conducted by Hanson et al. showed that high calorie supplements and other oral feeding interventions can help dementia patients with feeding problems gain weight. However, there was no impact of oral feeding interventions on physical function or mortality [143].

### 5.4. Enteral and Parenteral Nutrition

Enteral nutrition is recommended for patients who have a functional gastrointestinal system but are unable to consume their daily required calories over several days [144]. Enteral feeding is generally preferred to parenteral administration as it associated with better outcomes, including reduced inflammatory status, costs, nosocomial infections and mortality [145]. Parenteral nutrition should be considered when caloric needs are not met after 7 days of enteral feeding. Patients that need nutritional support and to whom enteral nutrition is contraindicated (e.g., intestinal obstruction, severe and recurring vomiting and diarrhoea, malabsorption, severe shock, peritonitis) may also benefit from parenteral infusion [146]. Enteral and parental nutrition should be used as the last solution in frail older people if deemed appropriate. There are important considerations to be made before deciding on the use of enteral and parental nutrition. These include patient preferences, and the prognosis and reversibility of the underlying cause of malnutrition. The effect of enteral nutrition on physical function, mortality and quality of life remains unclear [147,148,149,150]. Other important considerations include complications of enteral feeding such as aspiration pneumonia, agitation and self-extubation [151], and the burden of treatment faced by patients [149]. The ESPEN guideline recommends that the expected benefits and potential risks of enteral nutrition must be evaluated individually and reassessed regularly in particular when there is a change in clinical condition [3].

## 6. Conclusions

The projected increase in the number of older people with frailty underlines the importance of addressing malnutrition to improve the health of this group. The lack of an agreed standardised method to screen for malnutrition risk in older people across settings will continue to prove a challenge when attempting large-scale screening and treatment initiatives and calculating accurate prevalence rates. A complex mix of factors influence nutrition among older people but there are components of everyday practice within hospital and community settings that could be improved to address malnutrition. Clinicians have an important role in addressing these challenges by raising awareness of the issue of malnutrition among older people, developing care pathways and delivering training to key staff, such as primary care staff and healthcare students, to promote early intervention for older people with frailty who are at risk of malnutrition. In particular, studies addressing the impact of nutritional interventions on functional, clinical and patient-centered outcomes are needed.

## Figures and Tables

**Table 1:** Criteria for diagnosis of malnutrition. Diagnosis requires presence of one phenotypic and one aetiologic criteria [32]. BMI = body mass index.

**Phenotypic Criteria**	**Weight loss** >5% in last 6 months Or, >10% >6 months
	**Low BMI** <20 if <70 years Or, <22 if >70 years
	**Reduced muscle mass** (Using validated method of measurement)
**Etiologic Criteria**	**Reduced food intake or absorption** <50% of energy requirements Or, any reduction > 2 weeks Or, any chronic gastrointestinal condition impacting on absorption
	**Inflammation** Acute disease or injury Or, chronic disease states

**Table 2:** Global Leadership Initiative on Malnutrition guidance on grading of severity of malnutrition [32]. BMI = body mass index.

Grading of Malnutrition	Phenotypic Criteria		
	Weight Loss (%)	Low BMI	Low Muscle Mass
**Moderate**	5%–10% in last 6 months or 10%–20% >6 months	<20 if <70 years <22 if ≥70 years	Mild-to-moderate deficit
**Severe**	>10% in last 6 months or >20% >6 months	<18.5 if <70 years <20 if ≥70 years	Severe deficit
